# Supplementary material for: Itaconate promotes hepatocellular carcinoma progression by epigenetic induction of CD8+ T-cell exhaustion
Source: Nat Commun. 2023 Dec 9;14:8154. doi: 10.1038/s41467-023-43988-4 (PMC10710408; doi:10.1038/s41467-023-43988-4)
Supplement: Supplementary file 1 — Supplementary Information [file 41467_2023_43988_MOESM1_ESM.pdf]

Itaconate promotes hepatocellular carcinoma progression by epigenetic induction of CD8$^+$ T-cell exhaustion

Gu *et al.*

Inventory of Supporting Information

1. Supplementary Figures and figure legends

Supplementary Fig. 1 | Loss of IRG1 suppresses HCC progression *in vivo*.

Supplementary Fig. 2 | IRG1 drives CD8$^+$ T-cell-mediated tumor immune evasion**.**

Supplementary Fig. 3 | Macrophage-derived itaconate induces CD8$^+$ T-cell exhaustion.

Supplementary Fig. 4 | Itaconate induces CD8$^+$ T-cell exhaustion by promoting succinate-mediated H3K4me3 of *Eomes*.

Supplementary Fig. 5 | Ibuprofen inhibits HCC by blocking IRG1/itaconate-regulated immune evasion.

Supplementary Fig. 6 | Graphical abstract.

Supplementary Fig. 7 | Gating and sorting strategies.

Supplementary Fig. 8 | Individual analysis of t-SNE.

2. Supplementary Tables

Supplementary Table 1. qRT-PCR primer

Supplementary Table 2. ChIP-PCR primer

Supplementary Table 3. Reagent or resource information

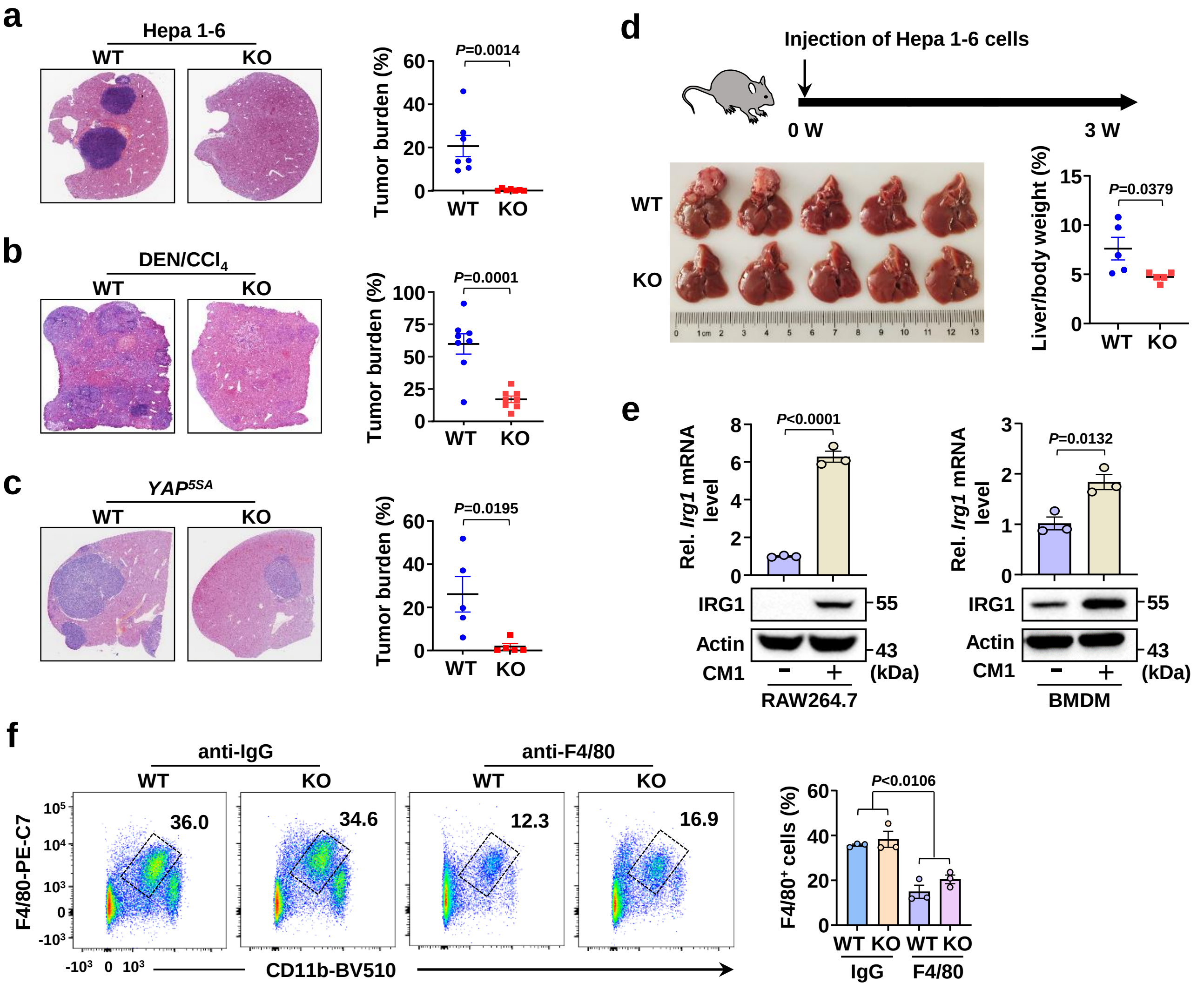

Supplementary Fig. 1 | Loss of IRG1 suppresses HCC progression *in vivo*. a Representative H&E staining of Fig. 1a is shown (left panel), and the tumor infiltration ratio was counted (right panel). Tumor burden (% of tumor area/total area). *n*=7 mice per group. **b** Representative H&E staining of Fig. 1b is shown (left panel), and the tumor infiltration ratio was counted (right panel). Tumor burden (% of tumor area/total area). *n*=8 mice per group. **c** Representative H&E staining of Fig. 1c is shown (left panel), and the tumor infiltration ratio was determined (right panel). Tumor burden (% of tumor area/total area). *n*=5 mice per group. **d** Schematic diagram of the Hepa 1-6 cell-induced HCC model (upper panel). WT and KO mice were injected with 1×10^5 Hepa 1-6 cells through the hepatic portal vein. Three weeks later, the female mice were euthanized. Liver images are shown, and the ratio of liver/body weight was measured (lower panel). *n*=5 female mice per group. **e** Analysis of IRG1 protein and *Irg1* mRNA levels in RAW264.7 cells and BMDMs treated with Hepa 1-6 cell supernatant (CM1) for 24 h. **f** Representative flow cytometry data and summary plot of the frequency showing the percentage of macrophages after anti-F4/80 antibody treatment of WT and KO mice with Hepa 1-6 cell-induced liver cancer. *n*=3 mice per group. All data represent mean ± SEM. Statistical significance was determined by unpaired two-tailed Student's *t*-test (**a**-**e**) and two-way ANOVA with Tukey's correction (**f**). Data are representative of three independent experiments with similar results (**e**). Source data are provided as a Source Data file.

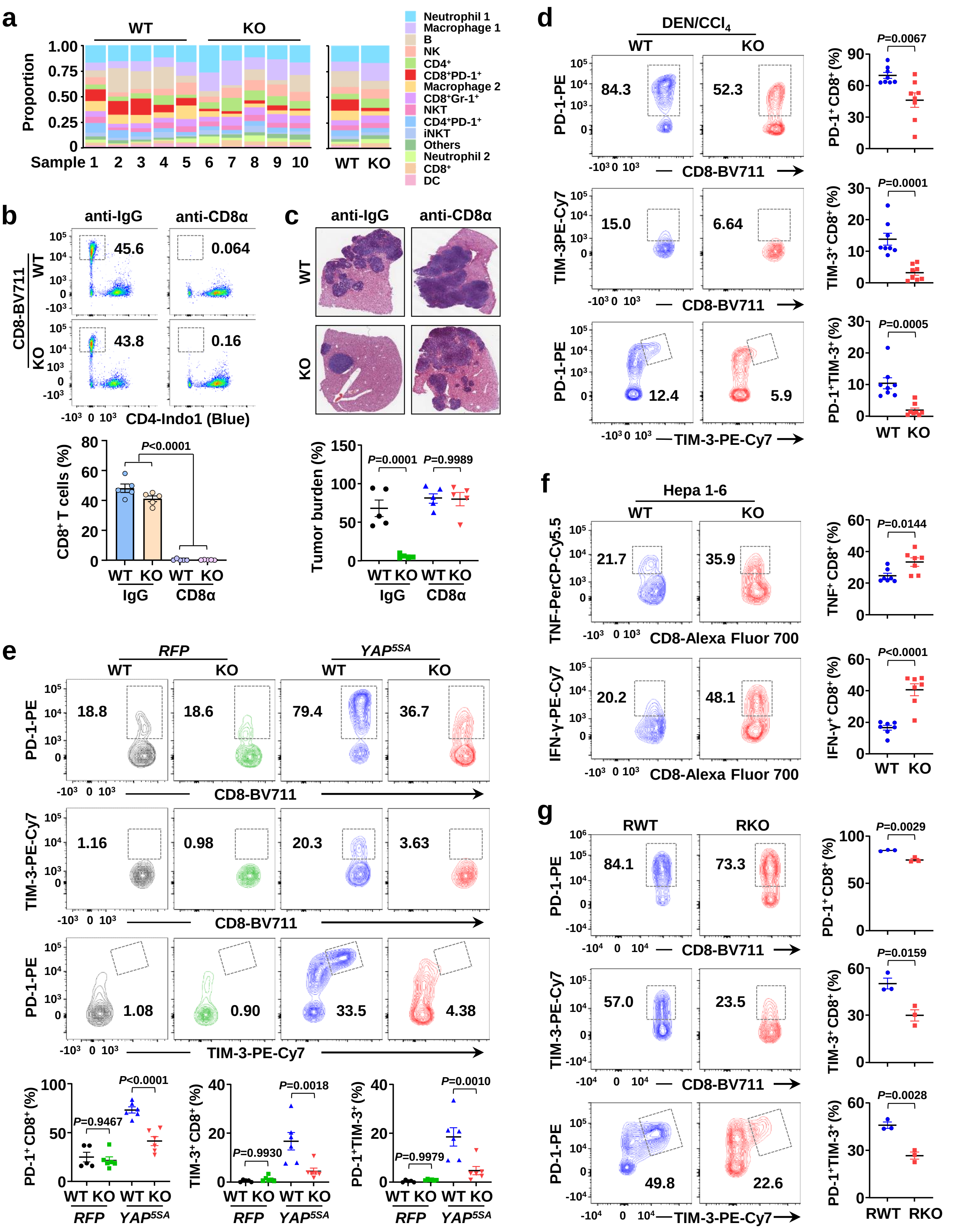

a
WT
KO
Proportion
Sample 1 2 3 4 5 6 7 8 9 10
WT KO
Neutrophil 1
Macrophage 1
B
NK
CD4+
CD8+PD-1+
Macrophage 2
CD8+Gr-1+
NKT
CD4+PD-1+
iNKT
Others
Neutrophil 2
CD8+
DC
b
anti-IgG
anti-CD8α
45.6
0.064
43.8
0.16
CD8-BV711
CD4-Indo1 (Blue)
CD8+ T cells (%)
P<0.0001
IgG
CD8α
c
anti-IgG
anti-CD8α
Tumor burden (%)
P=0.0001
P=0.9989
d
DEN/CCl4
84.3
52.3
PD-1-PE
CD8-BV711
PD-1+CD8+ (%)
P=0.0067
TIM-3PE-Cy7
15.0
6.64
TIM-3+CD8+ (%)
P=0.0001
12.4
5.9
TIM-3-PE-Cy7
PD-1+TIM-3+ (%)
P=0.0005
e
RFP
YAP5SA
18.8
18.6
79.4
36.7
1.16
0.98
20.3
3.63
1.08
0.90
33.5
4.38
PD-1+CD8+ (%)
P=0.9467
P<0.0001
TIM-3+CD8+ (%)
P=0.9930
P=0.0018
PD-1+TIM-3+ (%)
P=0.9979
P=0.0010
f
Hepa 1-6
TNF-PerCP-Cy5.5
21.7
35.9
CD8-Alexa Fluor 700
TNF+CD8+ (%)
P=0.0144
IFN-γ-PE-Cy7
20.2
48.1
IFN-γ+CD8+ (%)
P<0.0001
g
RWT
RKO
84.1
73.3
P=0.0029
57.0
23.5
P=0.0159
49.8
22.6
P=0.0028

Supplementary Fig. 2 | IRG1 drives $CD8^+$ T-cell-mediated tumor immune evasion. a Cell subsets of each sample defined by conventional manual analysis were projected onto a bar chart and assigned different colors from Fig. 2a. **b** Representative flow cytometry data (upper panel) and summary plot of the frequency (lower panel) showing the percentage of $CD8^+$ T cells after anti-CD8α antibody treatment in the Hepa 1-6 cell-induced HCC model of WT and KO mice. *n*=5 mice per group. **c** Representative H&E staining of Fig. 2b is shown (upper panel), and the corresponding tumor infiltration ratios were counted (lower panel). Tumor burden (% of tumor area/total area). *n*=5 mice per group. **d** Representative flow cytometry data (left panel) and summary plot (right panel) of the frequency showing the expression of $PD\text{-}1^+$, $TIM\text{-}3^+$, and $PD\text{-}1^+$ $TIM\text{-}3^+$ cells among $CD8^+$ TILs isolated from livers of WT and KO mice with DEN/CCl_4-induced liver cancer. *n*=8 mice per group. **e** Representative flow cytometry data and summary plot of the frequency showing the percentage of $PD\text{-}1^+$, $TIM\text{-}3^+$, and $PD\text{-}1^+$ $TIM\text{-}3^+$ cells among $CD8^+$ TILs isolated from livers of WT and KO mice with YAP^{5SA}-induced liver cancer. Red fluorescence protein (RFP) was used as a control. *n*=5 mice for WT (RFP), *n*=6 mice for KO (RFP), WT (YAP^{5SA}), and KO (YAP^{5SA}). **f** Representative flow cytometry data (left panel) and summary plot (right panel) of the frequency showing the expression of TNF^+ and IFN-γ^+ in $CD8^+$ TILs isolated from livers of WT and KO mice with Hepa 1-6 cell-induced liver cancer. *n*=7 mice per group. **g** Representative flow cytometry data (left panel) and summary plot (right panel) of the frequency showing the expression of $PD\text{-}1^+$, $TIM\text{-}3^+$, and $PD\text{-}1^+$ $TIM\text{-}3^+$ cells among $CD8^+$ T cells isolated from peripheral blood of Fig. 2g. *n*=3 mice per group. All data represent mean ± SEM. Statistical significance was determined by unpaired two-tailed Student's *t*-test (**d**, **f** and **g**) and two-way ANOVA with Tukey's correction (**b**, **c** and **e**). Source data are provided as a Source Data file.

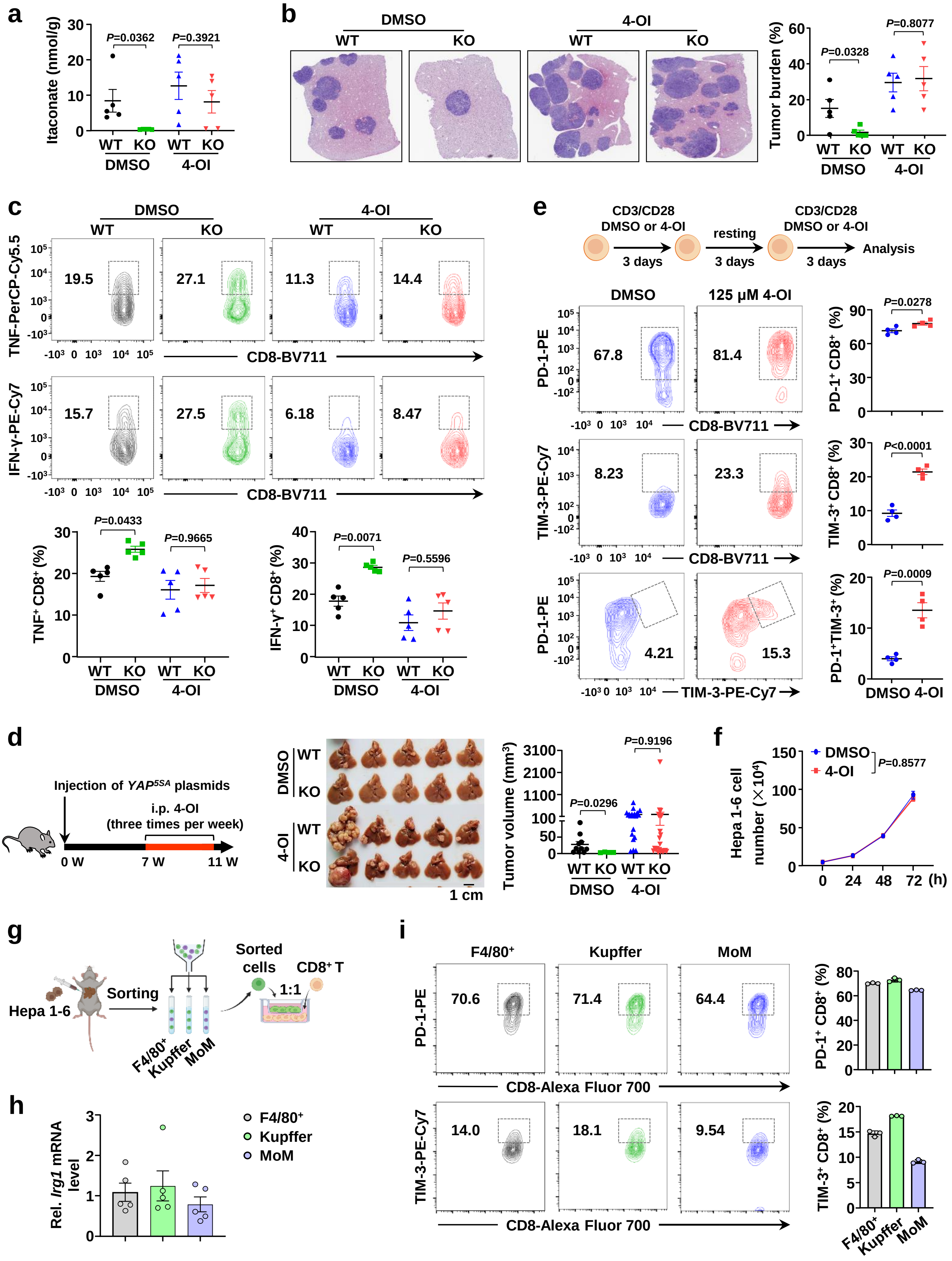

a
Itaconate (nmol/g)
P=0.0362
P=0.3921
WT KO WT KO
DMSO 4-OI
b
DMSO 4-OI
WT KO WT KO
Tumor burden (%)
P=0.0328
P=0.8077
c
DMSO 4-OI
WT KO WT KO
TNF-PerCP-Cy5.5
19.5
27.1
11.3
14.4
CD8-BV711
IFN-γ-PE-Cy7
15.7
27.5
6.18
8.47
TNF+ CD8+ (%)
P=0.0433
P=0.9665
IFN-γ+ CD8+ (%)
P=0.0071
P=0.5596
e
CD3/CD28 DMSO or 4-OI
3 days
resting
3 days
CD3/CD28 DMSO or 4-OI
3 days
Analysis
DMSO
125 μM 4-OI
PD-1-PE
67.8
81.4
TIM-3-PE-Cy7
8.23
23.3
4.21
15.3
TIM-3-PE-Cy7
PD-1+ CD8+ (%)
P=0.0278
TIM-3+ CD8+ (%)
P<0.0001
PD-1+TIM-3+ (%)
P=0.0009
DMSO 4-OI
d
Injection of YAP5SA plasmids
i.p. 4-OI
(three times per week)
0 W
7 W
11 W
DMSO
4-OI
1 cm
Tumor volume (mm3)
P=0.0296
P=0.9196
f
DMSO
4-OI
P=0.8577
Hepa 1-6 cell number (×10⁴)
0 24 48 72 (h)
g
Hepa 1-6
Sorting
Sorted cells
CD8+ T
1:1
F4/80+
Kupffer
MoM
h
Rel. Irg1 mRNA level
F4/80+
Kupffer
MoM
i
F4/80+
Kupffer
MoM
PD-1-PE
70.6
71.4
64.4
CD8-Alexa Fluor 700
TIM-3-PE-Cy7
14.0
18.1
9.54
PD-1+ CD8+ (%)
TIM-3+ CD8+ (%)

Supplementary Fig. 3 | Macrophage-derived itaconate induces CD8$^+$ T-cell exhaustion. a UPLC–MS/MS analysis of the itaconate abundance from mouse liver tissue in Fig. 3b. n=5 mice per group. **b** Representative H&E staining of Fig. 3b is shown (left panel), and the corresponding tumor infiltration ratio was counted (right panel). *n*=5 mice per group. **c** Representative flow cytometry data (upper panel) and summary plot of the frequency (lower panel) showing the expression of TNF$^+$ and IFN-γ$^+$ in CD8$^+$ TILs isolated from livers of WT and KO mice treated with control DMSO or 4-OI. *n*=5 mice per group. **d** Schematic diagram of *YAP*^{5SA}-induced liver cancer with 4-OI addition (left panel). Tumor images are shown (middle panel), and the tumor masses were measured at the end of the experiment (right panel). *n*=5 mice per group. **e** CD8$^+$ T cells were stimulated with plate-bound anti-CD3/CD28 and IL-2 in the presence or absence of 4-OI. Cells were then rested in the presence of IL-2 followed by restimulation with or without 4-OI. Representative flow cytometry data and summary plot of the frequency showing the percentage of PD-1$^+$, TIM-3$^+$, PD-1$^+$ TIM-3$^+$ cells among CD8$^+$ T cells. **f** Growth curves were measured in Hepa 1-6 cells treated with DMSO or 4-OI. **g** Schematic diagram of hepatic macrophage populations cocultured with CD8$^+$ T cells. **h** Analysis of *Irg1* mRNA levels in hepatic macrophage populations sorted from Hepa 1-6 cell-induced mouse liver. *n*=5 mice per group. **i** Representative flow cytometry data and summary plot showing the percentages of PD-1$^+$ and TIM-3$^+$ cells among CD8$^+$ T cells in Supplementary Fig. 3g. *n*=3 samples per group, cells from 5 mice were mixed as one sample. All data represent mean ± SEM. Statistical significance was determined by unpaired two-tailed Student's *t*-test (**a**, **b** and **e**), unpaired two-tailed Student's *t*-test with Welch's correction (**d**), one-way ANOVA (**f**) and two-way ANOVA with Tukey's correction (**c**). Data are representative of three independent experiments with similar results (**e**, **f**, **h** and **i**). Source data are provided as a Source Data file.

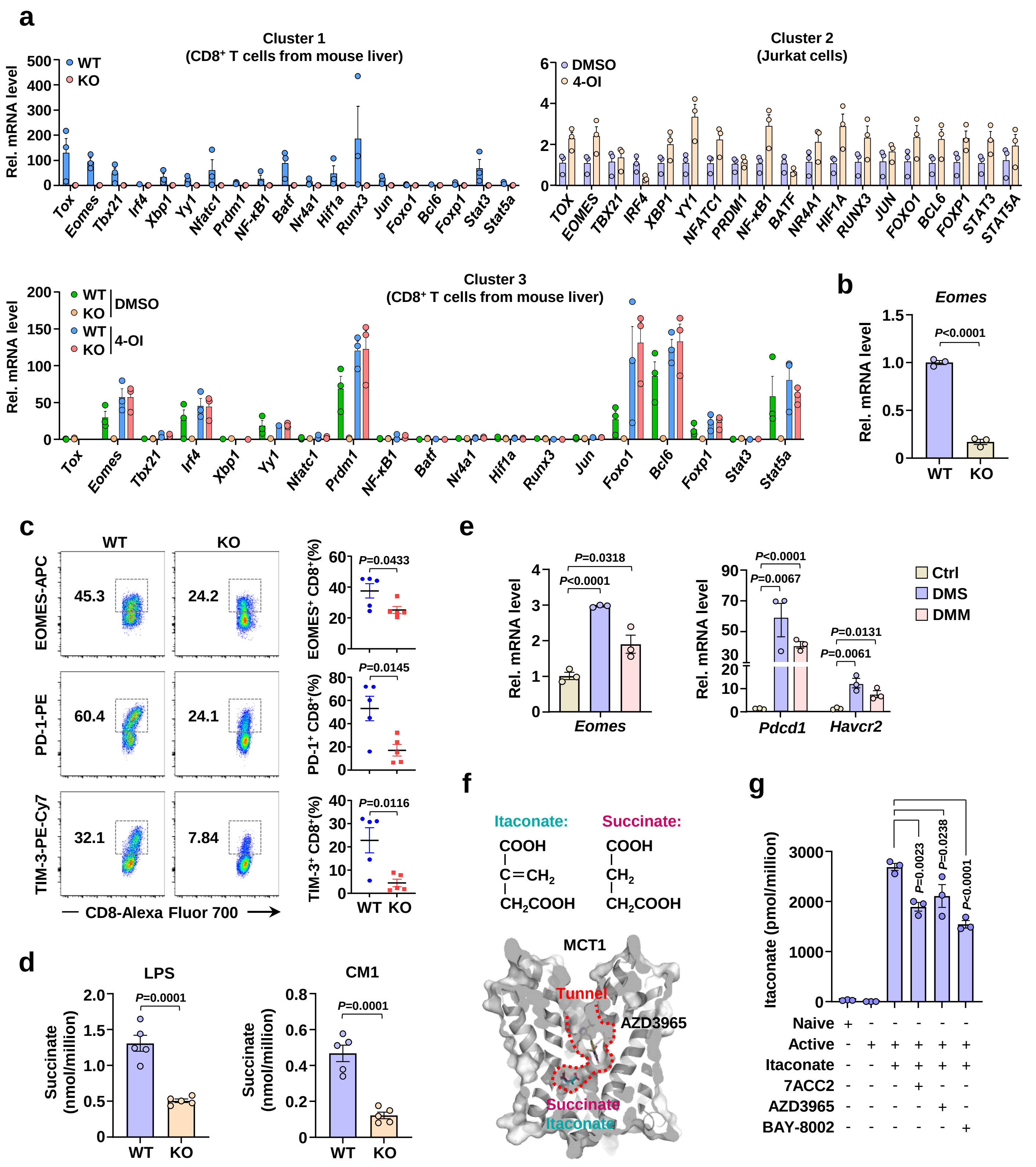

a
Cluster 1
(CD8+ T cells from mouse liver)
Cluster 2
(Jurkat cells)
Cluster 3
(CD8+ T cells from mouse liver)
Rel. mRNA level
WT
KO
DMSO
4-OI
Tox Eomes Tbx21 Irf4 Xbp1 Yy1 Nfatc1 Prdm1 NF-κB1 Batf Nr4a1 Hif1a Runx3 Jun Foxo1 Bcl6 Foxp1 Stat3 Stat5a
TOX EOMES TBX21 IRF4 XBP1 YY1 NFATC1 PRDM1 NF-κB1 BATF NR4A1 HIF1A RUNX3 JUN FOXO1 BCL6 FOXP1 STAT3 STAT5A
b
Eomes
P<0.0001
c
WT
KO
EOMES-APC
PD-1-PE
TIM-3-PE-Cy7
45.3
24.2
60.4
24.1
32.1
7.84
CD8-Alexa Fluor 700
EOMES+ CD8+(%)
PD-1+ CD8+(%)
TIM-3+ CD8+(%)
P=0.0433
P=0.0145
P=0.0116
d
LPS
CM1
Succinate (nmol/million)
P=0.0001
e
Ctrl
DMS
DMM
P=0.0318
P<0.0001
P=0.0067
P=0.0131
P=0.0061
Eomes
Pdcd1
Havcr2
f
Itaconate:
Succinate:
COOH
C=CH2
CH2COOH
COOH
CH2
CH2COOH
MCT1
Tunnel
AZD3965
Succinate
Itaconate
g
Itaconate (pmol/million)
P=0.0023
P=0.0238
P<0.0001
Naive
Active
Itaconate
7ACC2
AZD3965
BAY-8002

Supplementary Fig. 4 | Itaconate induces CD8⁺ T-cell exhaustion by promoting succinate-mediated H3K4me3 of *Eomes*. a Cluster 1: Analysis of the mRNA levels of exhausted TFs in $CD8^+$ TILs isolated from the Hepa 1-6 cell-induced HCC model. *n*=3 samples per group, 2 mice per sample. **Cluster 2:** Analysis of the mRNA levels of exhausted TFs in Jurkat cells stimulated with PHA in the presence of DMSO or 4-OI. **Cluster 3:** Analysis of the mRNA levels of exhausted TFs in $CD8^+$ TILs isolated from WT and KO mice i.p. treated with control DMSO or 4-OI after injection of Hepa 1-6 cells. *n*=3 samples per group, 2 mice per sample. **b** Analysis of the mRNA levels of *Eomes* in $CD8^+$ TILs isolated from the YAP^{5SA}-induced HCC model. *n*=3 samples per group, 2 mice per sample. **c** Representative flow cytometry data and summary plot showing the percentages of $EOMES^+$, PD-1^+, and TIM-3^+ cells among $CD8^+$ T cells in Supplementary Fig. 1d. *n*=5 female mice per group. **d** UPLC–MS/MS analysis of the intracellular succinate abundance in WT- and KO-derived BMDMs of Fig. 3a. *n*=5 samples per group. **e** Analysis of the mRNA levels of *Eomes*, *Pdcd1* and *Havcr2* in $CD8^+$ T cells treated with DMS or DMM. **f** Docking model of the wild type MCT1 (pdb:6lyy) bound to multiple ligands (AZD3965, succinate， itaconate) with visilization of transporter tunnel. AZD3965 (blue and yellow); Succinate (pink); Itaconate (cyan); MCT1 (white and grey). **g** UPLC–MS/MS analysis of the abundance of itaconate in the $CD8^+$ T cells. $CD8^+$ T cells isolated from WT mouse spleens were stimulated with plate-bound anti-CD3/CD28 and IL-2, recorded as “Active”. Unstimulated $CD8^+$ T cells were recorded as “Naïve”. Activated $CD8^+$ T cells were treated with MCT1 inhibitors for 30 min before itaconate treatment for 1 h. All data represent mean ± SEM. Statistical significance was determined by unpaired two-tailed Student’s *t*-test (**b**, **c**, **d**, and **e**) and one-way ANOVA with Tukey’s correction (**g**). Data are representative of three independent experiments with similar results (**e** and **g**). Source data are provided as a Source Data file.

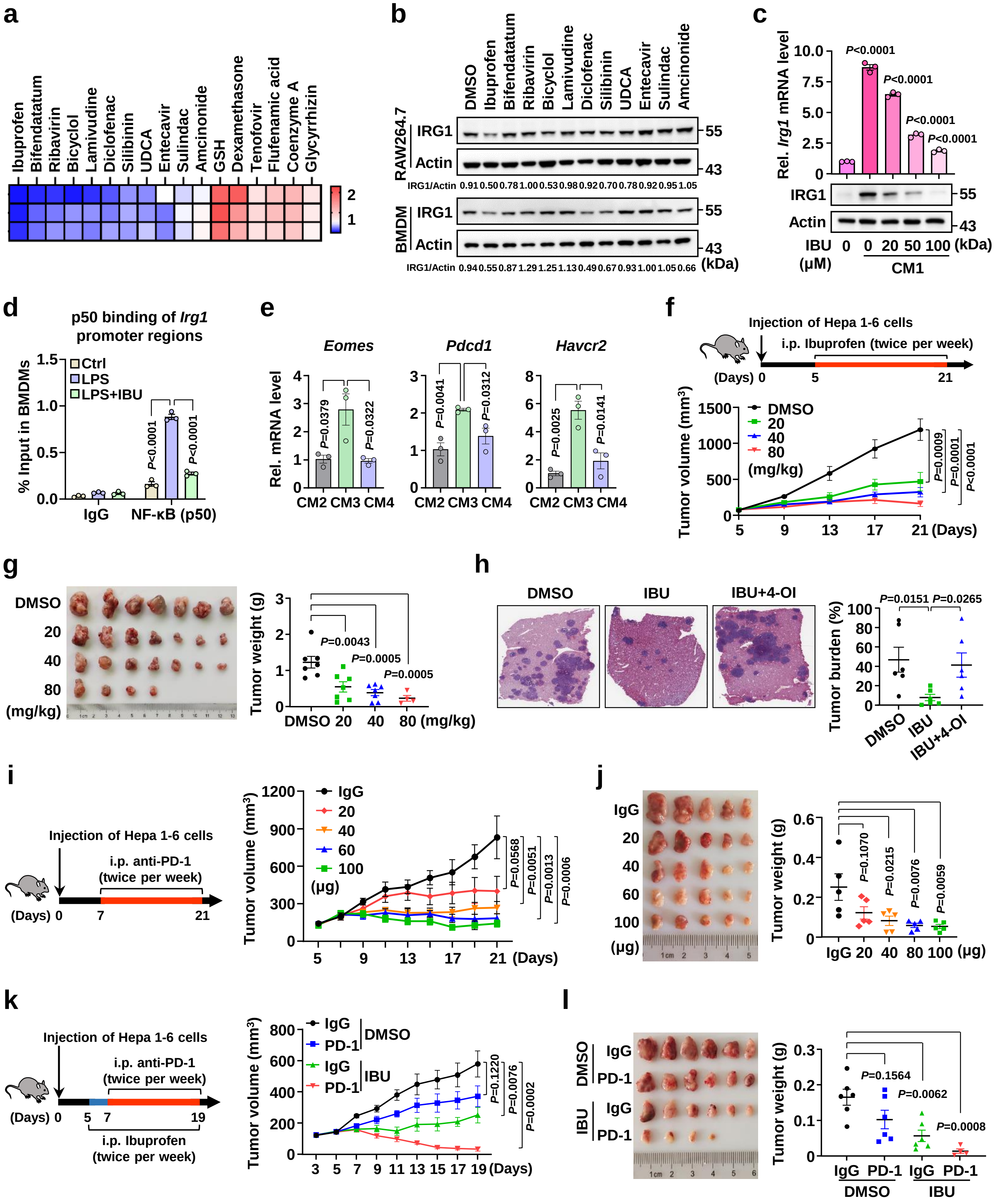
a
Ibuprofen
Bifendatatum
Ribavirin
Bicyclol
Lamivudine
Diclofenac
Silibinin
UDCA
Entecavir
Sulindac
Amcinonide
GSH
Dexamethasone
Tenofovir
Flufenamic acid
Coenzyme A
Glycyrrhizin
2
1
b
DMSO Ibuprofen Bifendatatum Ribavirin Bicyclol Lamivudine Diclofenac Silibinin UDCA Entecavir Sulindac Amcinonide
RAW264.7
IRG1
Actin
55
43
IRG1/Actin 0.91 0.50 0.78 1.00 0.53 0.98 0.92 0.70 0.78 0.92 0.95 1.05
BMDM
IRG1
Actin
IRG1/Actin 0.94 0.55 0.87 1.29 1.25 1.13 0.49 0.67 0.93 1.00 1.05 0.66
(kDa)
c
Rel. Irg1 mRNA level
P<0.0001
IRG1
Actin
IBU (μM) 0 0 20 50 100 (kDa)
CM1
d
p50 binding of Irg1 promoter regions
Ctrl
LPS
LPS+IBU
% Input in BMDMs
P<0.0001
IgG
NF-κB (p50)
e
Eomes
Pdcd1
Havcr2
Rel. mRNA level
P=0.0379
P=0.0322
P=0.0041
P=0.0312
P=0.0025
P=0.0141
CM2 CM3 CM4
f
Injection of Hepa 1-6 cells
i.p. Ibuprofen (twice per week)
(Days) 0 5 21
Tumor volume (mm³)
DMSO
20
40
80
(mg/kg)
P=0.0009
P<0.0001
P<0.0001
(Days)
g
DMSO
20
40
80
(mg/kg)
Tumor weight (g)
P=0.0043
P=0.0005
P=0.0005
DMSO 20 40 80 (mg/kg)
h
DMSO
IBU
IBU+4-OI
Tumor burden (%)
P=0.0151
P=0.0265
i
Injection of Hepa 1-6 cells
i.p. anti-PD-1 (twice per week)
(Days) 0 7 21
Tumor volume (mm³)
IgG
20
40
60
100
(μg)
P=0.0568
P=0.0051
P=0.0013
P=0.0006
(Days)
j
IgG
20
40
60
100
(μg)
Tumor weight (g)
P=0.1070
P=0.0215
P=0.0076
P=0.0059
IgG 20 40 80 100 (μg)
k
Injection of Hepa 1-6 cells
i.p. anti-PD-1 (twice per week)
(Days) 0 5 7 19
i.p. Ibuprofen (twice per week)
Tumor volume (mm³)
IgG
PD-1
DMSO
IgG
PD-1
IBU
P=0.1220
P=0.0076
P=0.0002
(Days)
l
DMSO
IgG
PD-1
IBU
IgG
PD-1
Tumor weight (g)
P=0.1564
P=0.0062
P=0.0008
IgG PD-1 IgG PD-1
DMSO IBU

Supplementary Fig. 5 | Ibuprofen inhibits HCC by blocking IRG1/itaconate-regulated immune evasion. a Analysis of *Irg1* mRNA levels in RAW264.7 cells treated with the indicated drugs. **b** Immunoblotting analysis of IRG1 protein levels in RAW264.7 cells and BMDMs stimulated with LPS and further treated with the indicated drugs. **c** Analysis of *Irg1* mRNA and protein levels in BMDMs treated with ibuprofen after Hepa 1-6 cell supernatant (CM1) stimulation. **d** ChIP experiments were performed in BMDMs treated with DMSO or ibuprofen using IgG or p50 antibodies. BMDMs were stimulated with LPS before ibuprofen treatment. The occupancy of potential binding sites in the *Irg1* gene by NF-κB (p50) was determined. **e** Analysis of *Eomes*, *pdcd1*, and *Havcr2* mRNA levels in $CD8^+$ T cells cultured with RAW264.7 cell supernatant of Fig. 5b. **f** WT mice were i.h. injected with Hepa 1-6 cells and were treated with ibuprofen. Tumor size was measured starting at Day 5 after inoculation. **g** Tumor images are shown (left panel), and the tumor masses were measured (right panel). *n*=7 mice per group. **h** Representative H&E staining of Fig. 5d is shown (left panel), and the tumor infiltration ratio was counted (right panel). *n*=6 mice per group. **i** WT mice were i.h. injected with Hepa 1-6 cells, and later they were treated with anti-PD-1 antibody. Tumor size was measured starting at Day 7 after inoculation. **j** Tumor images are shown, and the tumor masses were measured. *n*=5 mice per group. **k** WT mice were i.h. injected with Hepa 1-6 cells, and 5 days later, they were treated with ibuprofen or anti-PD-1 antibody. Tumor size was measured starting at Day 3 after inoculation. **l** Tumor images are shown, and the tumor masses were measured. *n*=6 mice per group. All data represent mean ± SEM. Statistical significance was determined by unpaired two-tailed Student's *t*-test (**h**), one-way ANOVA (**c**-**g**, **i** and **j**) and two-way ANOVA with Tukey's correction (**k**, **l**). Data are representative of three independent experiments with similar results (**a**-**e**). Source data are provided as a Source Data file.

a

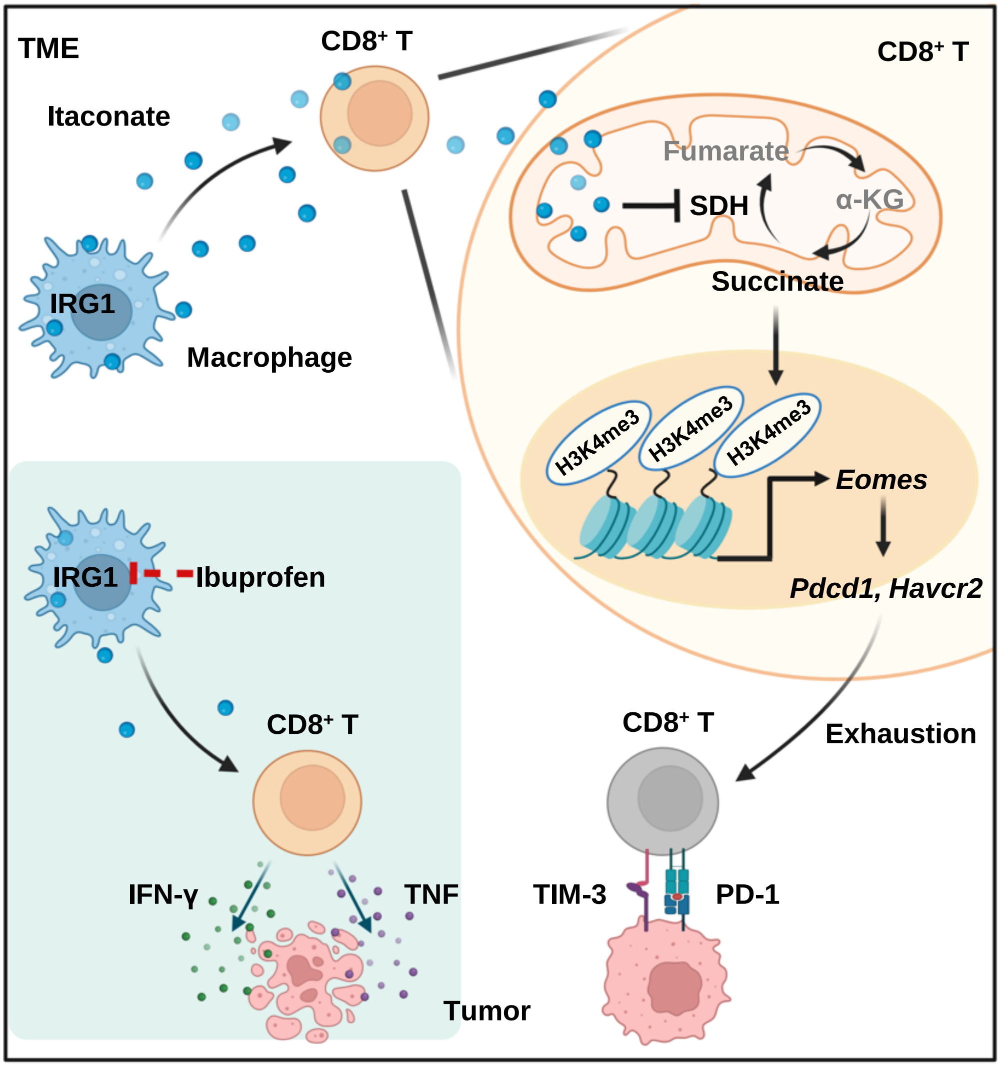

Supplementary Fig. 6 | Graphical abstract. a Working model of macrophage-derived itaconate induced CD8⁺ T-cell exhaustion.

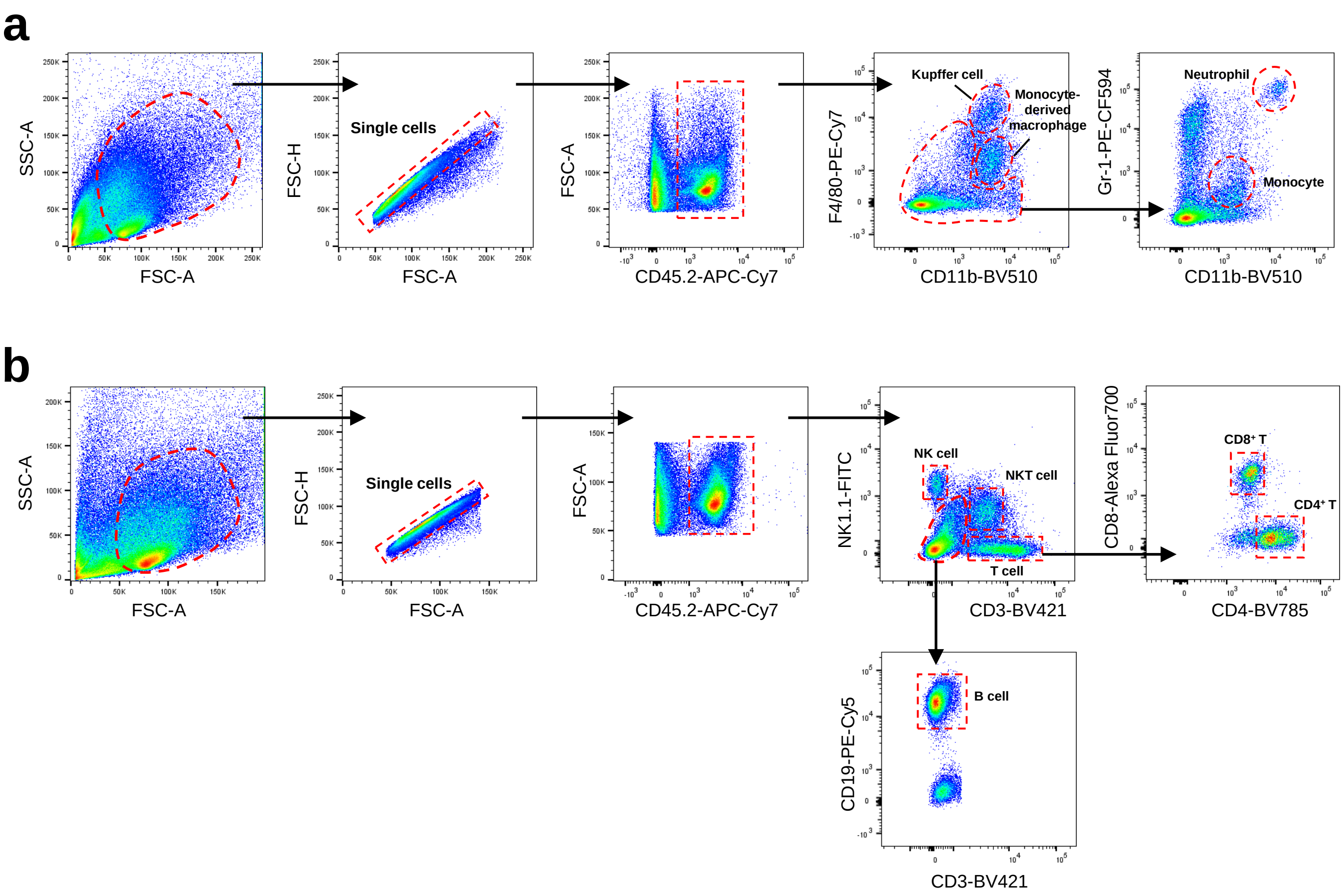

Supplementary Fig. 7 | **Gating and sorting strategies. a** Representative flow cytometry gating and sorting strategies of Kupffer cells, monocyte-derived macrophage, neutrophil and monocytes (Fig. 1d, Supplementary Fig. 1f and Supplementary Fig. 3g). **b** Representative flow cytometry gating and sorting strategies of NK, CD4^{+}T, CD8^{+}T and B cells. (Fig. 1d, 2c, 2d, 3c-e, 4b, 5b, 5f, 5i, Supplementary Fig. 2b, 2d-g, 3c, 3e, 3i and Supplementary Fig. 4c).

a

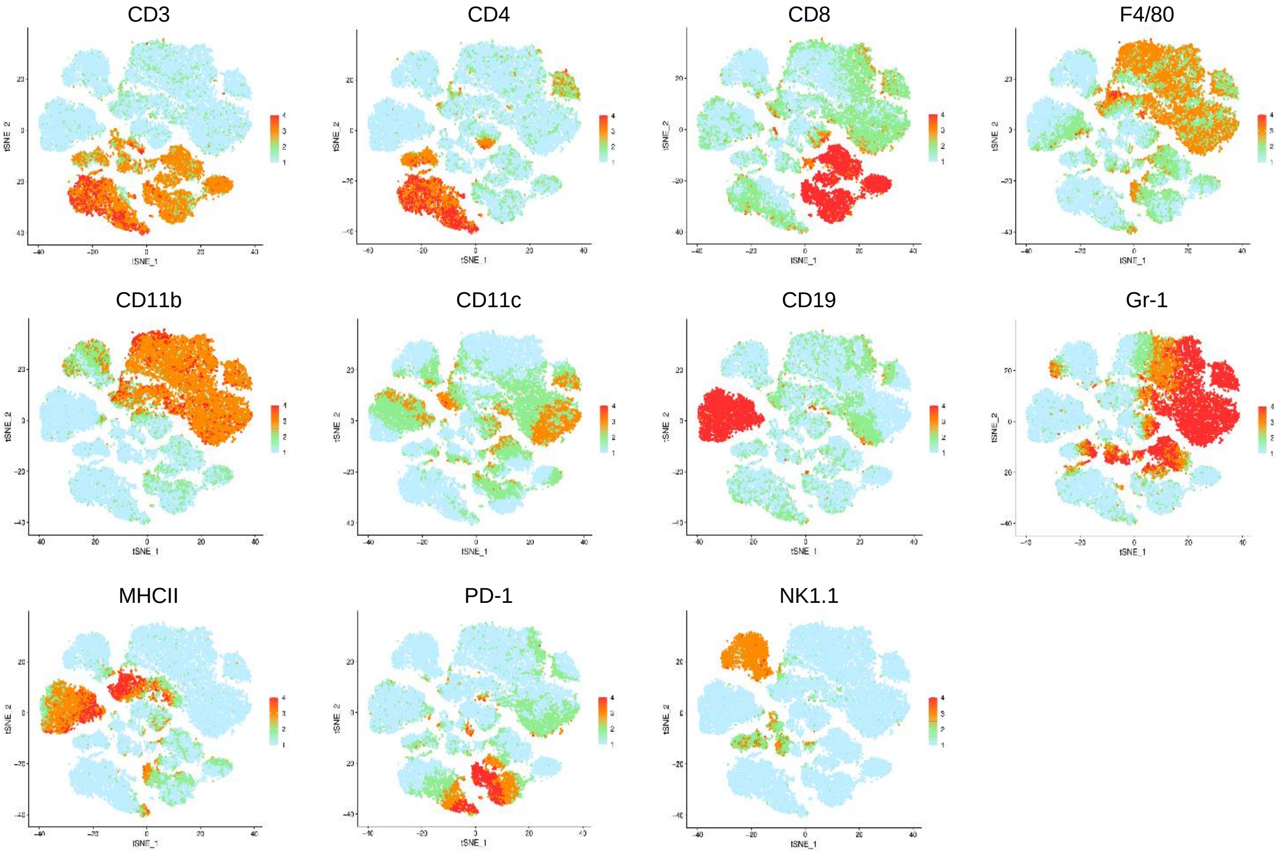

Supplementary Fig. 8 | **Individual analysis of t-SNE. a** t-SNE plot shows the projection of various immune cells. The functional description of each cluster shown in different colors is determined by the gene expression characteristics of each cluster (Fig. 2a).

Supplementary Table 1. qRT-PCR primer

Name	Sequence	Description
Prdm1-FWD	TGGAGGACGCTGATATGACTT	mouse
Prdm1-REV	GGGTGGTCGTTCACTATGTATG	mouse
Nfatc1-FWD	GACCCGGAGTTCGACTTCG	mouse
Nfatc1-REV	TGACACTAGGGGACACATAACTG	mouse
Batf-FWD	CTGGCAAACAGGACTCATCTG	mouse
Batf-REV	GGGTGTCGGCTTTCTGTGTC	mouse
Foxp1-FWD	GGTCTGAGACAAAAAGTAACGGA	mouse
Foxp1-REV	CGCACTCTAGTAAGTGGTTGC	mouse
NF-κB1-FWD	ATGGCAGACGATGATCCCTAC	mouse
NF-κB1-REV	TGTTGACAGTGGTATTTCTGGTG	mouse
Irf4-FWD	TCCGACAGTGGTTGATCGAC	mouse
Irf4-REV	CCTCACGATTGTAGTCCTGCTT	mouse
Jun-FWD	CCTTCTACGACGATGCCCTC	mouse
Jun-REV	GGTTCAAGGTCATGCTCTGTTT	mouse
Bcl6-FWD	CCGGCACGCTAGTGATGTT	mouse
Bcl6-REV	TGTCTTATGGGCTCTAAACTGCT	mouse
Foxo1-FWD	CCCAGGCCGGAGTTTAACC	mouse
Foxo1-REV	GTTGCTCATAAAGTCGGTGCT	mouse
Stat3-FWD	CAATACCATTGACCTGCCGAT	mouse
Stat3-REV	GAGCGACTCAAACTGCCCT	mouse
Stat5a-FWD	CGCCAGATGCAAGTGTTGTAT	mouse
Stat5a-REV	TCCTGGGGATTATCCAAGTCAAT	mouse
Nr4a1-FWD	TTGAGTTCGGCAAGCCTACC	mouse
Nr4a1-REV	GTGTACCCGTCCATGAAGGTG	mouse
Xbp1-FWD	AGCAGCAAGTGGTGGATTTG	mouse
Xbp1-REV	GAGTTTTCTCCCGTAAAAGCTGA	mouse
Runx3-FWD	CAGGTTCAACGACCTTCGATT	mouse
Runx3-REV	GTGGTAGGTAGCCACTTGGG	mouse
Yy1-FWD	CAGTGGTTGAAGAGCAGATCAT	mouse
Yy1-REV	AGGGAGTTTCTTGCCTGTCAT	mouse
Eomes-FWD	GCGCATGTTTCCTTTCTTGAG	mouse
Eomes-REV	GGTCGGCCAGAACCACTTC	mouse
Tbx21-FWD	AGCAAGGACGGCGAATGTT	mouse
Tbx21-REV	GGGTGGACATATAAGCGGTTC	mouse
Tox-FWD	GCTCCCGTTCCATCCACAAA	mouse
Tox-REV	TCCCAATCTCTTGCATCACAGA	mouse
Hif1a-FWD	ACCTTCATCGGAAACTCCAAAG	mouse
Hif1a-REV	CTGTTAGGCTGGGAAAAGTTAGG	mouse
Pdcd1-FWD	ACCCTGGTCATTCACTTGGG	mouse
Pdcd1-REV	CATTTGCTCCCTCTGACACTG	mouse
Havcr2-FWD	TCAGGTCTTACCCTCAACTGTG	mouse
Havcr2-REV	GGGCAGATAGGCATTTTTACCA	mouse
18S-FWD	CGCTACTACCGATTGGATGG	mouse
18S-REV	AGTTCGACCGTCTTCTCAGC	mouse
Actin-FWD	GGCTGTATTCCCCTCCATCG	mouse
Actin-REV	CCAGTTGGTAACAATGCCATGT	mouse
Irg1-FWD	GCAACATGATGCTCAAGTCTG	mouse
Irg1-REV	TGCTCCTCCGAATGATACCA	mouse

PRDM1-FWD	TAAAGCAACCGAGCACTGAGA	human
PRDM1-REV	ACGGTAGAGGTCCTTTCCTTTG	human
NFATC1-FWD	TGTGCCGGAATCCTGAAACTC	human
NFATC1-REV	GAGCATTCGATGGGGTTGGAG	human
BATF-FWD	TATTGCCGCCCAGAAGAGC	human
BATF-REV	GCTTGATCTCCTTGCGTAGAG	human
FOXP1-FWD	ATGATGCAAGAATCTGGGACTG	human
FOXP1-REV	GGATGGCTGAACCGTTACTTTT	human
NF-κB1-FWD	GAAGCACGAATGACAGAGGC	human
NF-κB1-REV	GCTTGGCGGATTAGCTCTTTT	human
IRF4-FWD	GCTGATCGACCAGATCGACAG	human
IRF4-REV	CGGTTGTAGTCCTGCTTGC	human
JUN-FWD	TCCAAGTGCCGAAAAAGGAAG	human
JUN-REV	CGAGTTCTGAGCTTTCAAGGT	human
BCL6-FWD	ACACATCTCGGCTCAATTTGC	human
BCL6-REV	AGTGTCCACAACATGCTCCAT	human
FOXO1-FWD	GGATGTGCATTCTATGGTGTACC	human
FOXO1-REV	TTTCGGGATTGCTTATCTCAGAC	human
STAT3-FWD	ACCAGCAGTATAGCCGCTTC	human
STAT3-REV	GCCACAATCCGGGCAATCT	human
STAT5A-FWD	GCAGAGTCCGTGACAGAGG	human
STAT5A-REV	CCACAGGTAGGGACAGAGTCT	human
NR4A1-FWD	ATGCCCTGTATCCAAGCCC	human
NR4A1-REV	GTGTAGCCGTCCATGAAGGT	human
XBP1-FWD	CCCTCCAGAACATCTCCCCAT	human
XBP1-REV	ACATGACTGGGTCCAAGTTGT	human
RUNX3-RWD	AGCACCACAAGCCACTTCAG	human
RUNX3-REV	GGGAAGGAGCGGTCAAACTG	human
YY1-FWD	CCTCTCAGATCCCAAACAACTG	human
YY1-REV	GCCTTTATGAGGGCAAGCTATT	human
EOMES-FWD	CTGCCCACTACAATGTGTTCG	human
EOMES-REV	GCGCCTTTGTTATTGGTGAGTTT	human
TBX21-FWD	GGTTGCGGAGACATGCTGA	human
TBX21-REV	GTAGGCGTAGGCTCCAAGG	human
TOX-FWD	TATGAGCATGACAGAGCCGAG	human
TOX-REV	GGAAGGAGGAGTAATTGGTGGA	human
HIF1A-FWD	CACCACAGGACAGTACAGGAT	human
HIF1A-REV	CGTGCTGAATAATACCACTCACA	human
PDCD1-FWD	ACGAGGGACAATAGGAGCCA	human
PDCD1-REV	GGCATACTCCGTCTGCTCAG	human
HAVCR2-FWD	TTGGACATCCAGATACTGGCT	human
HAVCR2-REV	CACTGTCTGCTAGAGTCACATTC	human
18S-FWD	CGGCGACGACCCATTCGAAC	human
18S-REV	GAATCGAACCCTGATTCCCCGTC	human

Supplementary Table 2. ChIP-PCR primer

Name	Sequence	Description
Negative contrl-FWD	GTTGGCAACTCTTGGAACCTT	mouse
Negative contrl-REV	GCACGCGCGAATTTGGAATA	mouse
Eomes-FWD	CTGGGACCTGCCAAACTAGA	mouse
Eomes-REV	TCTATGGCGCCGGAGAAAC	mouse
Irg1-FWD	GCTGCTCTGAGTGACTCCTT	mouse
Irg1-REV	TGCACACTCTCTTTACTGGGC	mouse
Negative contrl-FWD	CCCTGCATGTTATTGTCGGC	human
Negative contrl-REV	TCAATCCCACTGCCCACTAC	human
EOMES-FWD	TGGAAAGCTTCGCACTGTTC	human
EOMES-REV	TAAGCAATTACAGACGCCGC	human

Supplementary Table 3. Reagent or resource information

Reagent or resource	Source	Identifier
BB700 Rat Anti-Mouse TNF (clone MP6-XT22)	BD	Cat:# 566510
PE/Cyanine7 anti-mouse IFN-γ (clone XMG1.2)	Biolegend	Cat:# 505826
PE/Cyanine7 anti-mouse CD366 (Tim-3) (clone RMT3-23)	Biolegend	Cat:# 119716
Brilliant Violet 421™ anti-mouse CD3 (clone 17A2)	Biolegend	Cat:# 100228
Brilliant Violet 711™ anti-mouse CD8a (clone 53-6.7)	Biolegend	Cat:# 100759
PE anti-mouse CD279 (PD-1) (clone 29F.1A12)	Biolegend	Cat:# 135206
BV510 Hamster Anti-Mouse CD3e (clone 145-2C11)	BD	Cat:# 563024
BUV563 Rat Anti-Mouse CD4 (clone GK1.5)	BD	Cat:# 612923
FITC anti-mouse NK-1.1 (clone PK136)	Biolegend	Cat:# 108706
Brilliant Violet 650™ anti-mouse NK-1.1 (clone PK136)	Biolegend	Cat:#108736
APC/Cyanine7 anti-mouse CD45.2 (clone 104)	Biolegend	Cat:# 109824
PerCP-Cy™5.5 Hamster Anti-Mouse CD11c (clone HL3)	BD	Cat:# 560584
PE-CF594 Rat Anti-mouse Ly-6G and Ly-6C (clone RB6-8C5)	BD	Cat:# 562710
PE/Cyanine7 anti-mouse F4/80 Antibody (clone BM8)	Biolegend	Cat:# 123114
APC anti-mouse Eomes Antibody (clone Dan11mag)	Invitrogen	Cat:# 17-4875-80
BV785 Rat anti-mouse CD8α (clone 53-6.7)	Biolegend	Cat:# 100750
Alexa Fluor® 700 anti-mouse CD8α (clone 53-6.7)	Biolegend	Cat:# 100730
V500 Rat anti-CD11b (clone M1/70)	BD	Cat:# 562127
Alexa Fluor® 700 anti-mouse I-A/I-E Antibody (MHC II) (clone M5/114.15.2)	Biolegend	Cat:# 107622
PE/Cyanine5 anti-mouse CD19 Antibody (clone 6D5)	Biolegend	Cat:# 115510
Purified anti-mouse CD28 (clone E18)	Biolegend	Cat:# 122002
Purified anti-mouse CD3ε (clone 145-2C11)	Biolegend	Cat:# 100302
InvivoMAb anti-mouse CD8α (clone 2.43)	BioXcell	Cat:# BE0061
InvivoMAb anti-mouse F4/80 (clone C1:A3-1)	BioXcell	Cat:# BE0206
InvivoMAb rat IgG2b isotype control (clone LTF-2)	BioXcell	Cat:# BE0090
InvivoMAb rat IgG2a isotype control (clone 2A3)	BioXcell	Cat:# BE0089
InvivoMAb anti-mouse PD-1 (CD279) (clone RMP1-14)	BioXcell	Cat:# BE0146

Anti-Histone H3 (mono methyl K4) (clone ERP16597)	Abcam	Cat:# ab176877
Anti-Histone H3 (di methyl methyl K4) (clone Y47)	Abcam	Cat:# ab32356
Anti-Histone H3 (tri methyl methyl K4) (clone EPR20551-225)	Abcam	Cat:# ab213224
Anti-Histone H3 (mono methyl methyl K9) (clone EPR16989)	Abcam	Cat:# ab176880
Anti-Histone H3 (di methyl methyl K9) (clone EP16990)	Abcam	Cat:# ab176882
Anti-Histone H3 (tri methyl methyl K9) (clone EPR16601)	Abcam	Cat:# ab176916
Anti-Histone H3 (tri methyl methyl K27) (clone EPR18607)	Abcam	Cat:# ab192985
Histone-3 polyclonal antibody	proteintech	Cat:# 17168-1-AP
Eomes/TBR2 polyclonal antibody	proteintech	Cat:# 28316-1-AP
Anti-IRG1 (clone EPR22066)	Abcam	Cat:# ab222411
NF-κB1 p105/p50 (D4P4D) Rabbit mAb	Cell Signaling	Cat:# 13586
AZD3965	SelleckChem	Cat:# S7339
BAY-8002	SelleckChem	Cat:# S8747
7ACC2	SelleckChem	Cat:# S6732
RPMI Medium 1640 basic (1×)	Gibco	Cat:# C11875500BT
DMEM	Vivacell	Cat:# 2211070
Fetal Bovine Serum	Gibco	Cat:#10099141
Percoll	Cytiva	Cat:# 17089109
4-Octyl itaconate	Targetmol	Cat:# T4580
Phytohemagglutinin PHA-P	Sigma	Cat:# L9017
LPS	Sigma	Cat:# L2630
Cocktail	Sigma	Cat:#5056489001
Cell stimulation cocktail plus protein transport inhibitors	Invitrogen	Cat:#00497593
2-Mercaptoethanol	Gibco	Cat:# 21985-023
Red Blood Cell Lysis Buffer	Beyotime	Cat:# C3702
HEPES (1 M)	Gibco	Cat:# 15630-080
Penicillin-Streptomycin Solution	Biological Industries	Cat:# 03-031-1
GlutaMAX™	Gibco	Cat:# 35050-061
Sodium Pyruvate	Gibco	Cat:# 11360-070
Ringer's solution	LEAGENE	Cat:# CZ0045
Carbon tetrachloride (CCl_4)	MACKLIN	Cat:# C805329
N-Nitrosodiethylamine (DEN)	Sigma	Cat:# N0756
Murine IL-2	proteintech	Cat:# 212-12
Dimethyl succinate (DMS)	Sigma	Cat:# 73605
Dimethyl malonate (DMM)	Targetmol	Cat:# T5695
Olive oil	MACKLIN	Cat:# C13288835
Collagenase IV	Sigma	Cat:# C5138
Ibuprofen	Targetmol	Cat:# T1394
Bifendatatum	Targetmol	Cat:# T3273
Ribavirin	Targetmol	Cat:# T0684
Bicyclol	Targetmol	Cat:# T4121
Lamivudine	Targetmol	Cat:# T0682
Diclofenac	Targetmol	Cat:# T0196
Silibinin	Targetmol	Cat:# T1660
Ursodeoxycholic acid (UDCA)	Targetmol	Cat:# T0700
Entecavir	Targetmol	Cat:# T0085L
Sulindac	Targetmol	Cat:# T0459
Amcinonide	Targetmol	Cat:# T0261
GSH	Targetmol	Cat:# T1085
Dexamethasone	Targetmol	Cat:# T1076
Tenofovir	Targetmol	Cat:# T1649
Flufenamic acid	Targetmol	Cat:# T0858
Coenzyme A	Targetmol	Cat:# T10857
Glycyrrhizin	Targetmol	Cat:# T2741
CD4+ T Cell Isolation Kit mouse	Miltenyi Biotec	Cat:# 130-104-454
CD8 (TIL) MicroBeads mouse	Miltenyi Biotec	Cat:# 130-116-478
CD8α+ T Cell Isolation Kit mouse	Miltenyi Biotec	Cat:# 130-104-075
Succinate assay	Abcam	Cat:# ab204718
Alpha Ketoglutarate (alpha KG) assay	Abcam	Cat:# 83431
Fumarate assay	Abcam	Cat:# ab102516
ChIP assay kit	Beyotime	Cat:# P2078
