# Supplementary material for: Itaconate promotes hepatocellular carcinoma progression by epigenetic induction of CD8+ T-cell exhaustion
Source: Nat Commun. 2023 Dec 9;14:8154. doi: 10.1038/s41467-023-43988-4 (PMC10710408; doi:10.1038/s41467-023-43988-4)
Supplement: Supplementary file 3 — Reporting Summary [file 41467_2023_43988_MOESM3_ESM.pdf]

nature portfolio

Corresponding author(s): Ping Gao

Last updated by author(s): Oct 7, 2023

Reporting Summary

Nature Portfolio wishes to improve the reproducibility of the work that we publish. This form provides structure for consistency and transparency in reporting. For further information on Nature Portfolio policies, see our Editorial Policies and the Editorial Policy Checklist.

Statistics

For all statistical analyses, confirm that the following items are present in the figure legend, table legend, main text, or Methods section.

n/a	Confirmed	
☐	☒	The exact sample size (*n*) for each experimental group/condition, given as a discrete number and unit of measurement
☐	☒	A statement on whether measurements were taken from distinct samples or whether the same sample was measured repeatedly
☐	☒	The statistical test(s) used AND whether they are one- or two-sided<br>*Only common tests should be described solely by name; describe more complex techniques in the Methods section.*
☒	☐	A description of all covariates tested
☐	☒	A description of any assumptions or corrections, such as tests of normality and adjustment for multiple comparisons
☐	☒	A full description of the statistical parameters including central tendency (e.g. means) or other basic estimates (e.g. regression coefficient) AND variation (e.g. standard deviation) or associated estimates of uncertainty (e.g. confidence intervals)
☐	☒	For null hypothesis testing, the test statistic (e.g. *F*, *t*, *r*) with confidence intervals, effect sizes, degrees of freedom and *P* value noted<br>*Give P values as exact values whenever suitable.*
☒	☐	For Bayesian analysis, information on the choice of priors and Markov chain Monte Carlo settings
☒	☐	For hierarchical and complex designs, identification of the appropriate level for tests and full reporting of outcomes
☒	☐	Estimates of effect sizes (e.g. Cohen's *d*, Pearson's *r*), indicating how they were calculated

Our web collection on statistics for biologists contains articles on many of the points above.

Software and code

Policy information about availability of computer code

Data collection	Ultra-performance liquid chromatography coupled to a tandem-mass spectrometry (UPLC–MS/MS, ACQUITY UPLC-Xevo TQ-S, Waters Corp., Milford, MA, USA) system was used to collect metabolic data. Images were captured using a Leica microscope (Aperio CS2).
Data analysis	Masslynx (v4.1, Waters, Milford, MA, USA) and iMAP (v1.0, Metabo-Profile, Shanghai, China) were used to analyze metabolic data.<br>t-SNE analysis was performed using the R language (Version 4.0.5), Seurat package (version 4.0.2), and Flowjo software (10.8.1).<br>TCGA LIHC gene expression analysis was performed using the R (version 4.0.5), survival R package (version 3.2-13), survminer R package (version 0.4.9), and survMisc R package (version 0.5.5).<br>H&E images were performed using HALO (v3.3) software.

For manuscripts utilizing custom algorithms or software that are central to the research but not yet described in published literature, software must be made available to editors and reviewers. We strongly encourage code deposition in a community repository (e.g. GitHub). See the Nature Portfolio guidelines for submitting code & software for further information.

Data

Policy information about availability of data

All manuscripts must include a data availability statement. This statement should provide the following information, where applicable:

- Accession codes, unique identifiers, or web links for publicly available datasets
- A description of any restrictions on data availability
- For clinical datasets or third party data, please ensure that the statement adheres to our policy

This paper does not report any original code. TCGA LIHC gene expression and survival data were downloaded from https://www.cancer.gov/abouut-nci/organization/ccg/research/structural-genomics/tcga.

Research involving human participants, their data, or biological material

Policy information about studies with human participants or human data. See also policy information about sex, gender (identity/presentation), and sexual orientation and race, ethnicity and racism.

Reporting on sex and gender	N/A
Reporting on race, ethnicity, or other socially relevant groupings	N/A
Population characteristics	N/A
Recruitment	N/A
Ethics oversight	N/A

Note that full information on the approval of the study protocol must also be provided in the manuscript.

Field-specific reporting

Please select the one below that is the best fit for your research. If you are not sure, read the appropriate sections before making your selection.

- [x] Life sciences
- [ ] Behavioural & social sciences
- [ ] Ecological, evolutionary & environmental sciences

For a reference copy of the document with all sections, see nature.com/documents/nr-reporting-summary-flat.pdf

Life sciences study design

All studies must disclose on these points even when the disclosure is negative.

Sample size	We determined the sample sizes based on preliminary studies in our laboratories or in similarly published research (PMID: 35121990, PMID: 36864172, PMID: 36376563). The samples were enough to be detected and we saw statistical significant difference in replicated independent experiments. For animal experiments, we used at least 3 C57BL/6 mice per group and followed the 3 R's of animal research.
Data exclusions	No data were excluded from the data set.
Replication	We defined each sample in different groups performing three independent biological experiments, and the results showed the same trend. For animal studies, we used at least 3 C57BL/6 mice for different groups, and the statistical significance was shown in figures. We confirmed successful replication for our reported data.
Randomization	Mice were randomly allocated to control group or treatment groups. In vitro studies, cells or conditions were assigned randomly to each experimental group.
Blinding	Before performing Hematoxylin-eosin staining (H&E), the researchers took the same spot in the mouse liver to rule out potential bias caused by subjective selection of tumor sites. Investigators were blinded for H&E analysis. In other experiments, as detailed in the Methods section, samples were analyzed in a blinded manner without subjective estimation.

Reporting for specific materials, systems and methods

We require information from authors about some types of materials, experimental systems and methods used in many studies. Here, indicate whether each material, system or method listed is relevant to your study. If you are not sure if a list item applies to your research, read the appropriate section before selecting a response.

Materials & experimental systems

n/a	Involved in the study
	☒ Antibodies
	☒ Eukaryotic cell lines
☒	☐ Palaeontology and archaeology
	☒ Animals and other organisms
☒	☐ Clinical data
☒	☐ Dual use research of concern
☒	☐ Plants

Methods

n/a	Involved in the study
☒	☐ ChIP-seq
	☒ Flow cytometry
☒	☐ MRI-based neuroimaging

Antibodies

Antibodies used

Antibodies for flow cytometry:
BB700-TNF, clone MP6-XT22, BD, Cat:# 566510, Lot:# 9289992, 1:100
PE/Cyanine7-IFN-γ, clone XMG1.2, Biolegend, Cat:# 505826, Lot# B313721, 1:100
PE/Cyanine7-Tim-3, clone RMT3-23, Biolegend, Cat:# 119716, Lot# B325363, 1:100
Brilliant Violet 421-CD3, clone 17A2, Biolegend, Cat:# 100228, Lot# B317392, 1:100
Brilliant Violet 711-CD8a, clone 53-6.7, Biolegend, Cat:# 100759, Lot# B265869, 1:100
PE-PD-1, clone 29F.1A12, Biolegend, Cat:# 135206, Lot# B330031, 1:100
BV510-CD3e, clone 145-2C11, BD, Cat:# 563024, Lot# 1155692, 1:100
BUV563-CD4, clone GK1.5, BD, Cat:# 612923, Lot# 1088523, 1:100
FITC-NK1.1, clone PK136, Biolegend, Cat:# 108706, Lot# B323640, 1:100
Brilliant Violet 650-NK1.1, clone PK136, Biolegend, Cat:#108736, Lot#1303290
APC/Cyanine7-CD45.2, clone 104, Biolegend, Cat:# 109824, Lot# B338088, 1:100
PerCP-Cy™5.5-CD11c, clone HL3, BD, Cat:# 560584, Lot# 1067094, 1:100
PE-CF594-Ly-6G and Ly-6C, clone RB6-8C5, BD, Cat:# 562710, Lot# 1124024, 1:100
PE/Cyanine7-F4/80, clone BM8, Biolegend, Cat:# 123114, Lot# B335043, 1:100
APC-EOMES, clone Dan11mag, Invitrogen, Cat:# 17-4875-80, Lot:# 2473687, 1:100
BV785-CD8a, clone 53-6.7, Biolegend, Cat:#100750 , Lot# B285904, 1:100
Alexa Fluor® 700-CD8, clone 53-6.7, Biolegend, Cat:# 100730, Lot:# B360990, 1:100
V500-CD11b, clone M1/70, BD, Cat:# 562127, Lot# 9239901, 1:100
Alexa Fluor® 700-I-A/I-E, clone M5/114.15.2, Biolegend, Cat:# 107622, Lot# B264454, 1:100
PE/Cyanine5-CD19, clone 6D5, Biolegend, Cat:# 115510, Lot# B288417, 1:100
CD16/CD32 Monoclonal Antibody, clone 93, Biolegend, Cat:# 101302, Lot:#B320249, 1:100
Antibodies for injection of mice:
InvivoMAb anti-mouse F4/80 ,clone A3-1,   BioXcell, Cat:# BE0206, Lot:# 742620O1
InvivoMAb anti-mouse CD8a, clone 2.43, BioXcell, Cat:# BE0061, Lot:# 732021M1
InvivoMAb anti-mouse Ig2a isotype control, clone 2A3, BioXcell, Cat:# BE0089, Lot:# 796721M2
InvivoMAb anti-mouse Ig2b isotype control, clone LTF-2, BioXcell, Cat:# BE0090, Lot:# 767920D1
InvivoMAb anti-mouse PD-1, clone RMP1-14, BioXcell, Cat:# BE0146, Lot:# 810421N1
Antibodies for CD8+ T cell activation:
Purified-CD3ε, clone 145-2C11, Biolegend, Cat:# 100302, Lot:# B341584
Purified-CD28, clone E18, Biolegend, Cat:# 122002, Lot:# B370362
Antibodies for western blot:
H3K4me1 monoclonal antibody, clone ERP16597, Abcam, Cat:# ab176877, Lot# GR3208750-3, 1:1000
H3K4me2 monoclonal antibody, clone Y47, Abcam, Cat:# ab32356, Lot# GR3422075-1, 1:1000
H3K4me3 monoclonal antibody, clone EPR20551-225, Abcam, Cat:# ab213224, Lot# GR3213864-3, 1:1000, 1:50 For ChIP
H3K9me1 monoclonal antibody, clone EPR16989, Abcam, Cat:# ab176880, Lot# GR196241-14, 1:1000
H3K9me2 monoclonal antibody, clone EP16990, Abcam, Cat:# ab176882, Lot# GR3390355-6, 1:1000
H3K9me3 monoclonal antibody, clone EPR16601, Abcam, Cat:# ab176916, Lot# GR3218257-11, 1:1000
H3K27me3 monoclonal antibody, clone EPR18607, Abcam, Cat:# ab192985, Lot# GR3264827-13, 1:1000
Histone-3 polyclonal antibody, Proteintech, Cat:# 17168-1-AP, Lot# 00094157, 1:1000
Eomes/TBR2 polyclonal antibody, Proteintech, Cat:# 28316-1-AP, Lot# 71111, 1:1000
IRG1 monoclonal antibody, clone EPR22066, Abcam, Cat:# ab222411, Lot# GR3315968-2, 1:1000
NF-κB1 p105/p50 Rabbit mAb, clone D4P4D, Cell Signaling, Cat:# 13586, Lot# 5, 1:50 for ChIP

Validation

These antibodies have either been validated in published literatures or validated in our lab by Western blot using specific targeting shRNAs or over-expressing vectors. Validation statements were shown on the maufactures's website, including Biolegend, BD, Abcam, Proteintech, BioXcell, and Invitrogen.

Eukaryotic cell lines

Policy information about cell lines and Sex and Gender in Research

Cell line source(s)	Hepa 1-6, Jurkat (E6-1), and RAW264.7 cells were purchased from ATCC.
Authentication	Cell line identities were confirmed by STR fingerprinting.
Mycoplasma contamination	All cell lines were tested routinely to make sure they are negative for mycoplasma contamination by Mycoplasma PCR.
Commonly misidentified lines (See ICLAC register)	No commonly misidentified cell lines were used.

Animals and other research organisms

Policy information about studies involving animals; ARRIVE guidelines recommended for reporting animal research, and Sex and Gender in Research

Laboratory animals	Irg1-/- mice (C57BL/6NJ-Acod1em1(IMPC)J/J, stock #029340) were purchased from the Jackson Laboratory. Rag1-/- mice (strain No. T004753) were purchased from GemPharmatech (Nanjing, China). C57BL/6 mice were purchased from SLAC animal company. Six- to eight-week-old mice were used for all animal experiments unless YAP5SA-induced 4-week-old mice and DEN/CCl4-induced 2-week-old mice. These mice were maintained in 12 h light/ 12 h dark cycle, and the housing temperature and humidity were maintained were 24℃ and 50%, respectively.
Wild animals	Not used.
Reporting on sex	Sex-independent
Field-collected samples	No field-collected samples were used in this study.
Ethics oversight	All animal studies were conducted with approval from the Animal Research Ethics Committee of South China University of Technology.

Note that full information on the approval of the study protocol must also be provided in the manuscript.

Flow Cytometry

Plots

Confirm that:

- [x] The axis labels state the marker and fluorochrome used (e.g. CD4-FITC).
- [x] The axis scales are clearly visible. Include numbers along axes only for bottom left plot of group (a 'group' is an analysis of identical markers).
- [x] All plots are contour plots with outliers or pseudocolor plots.
- [x] A numerical value for number of cells or percentage (with statistics) is provided.

Methodology

Sample preparation	C57BL/6 mouse liver tissues were digested in the presence of collagenase IV prior to density gradient centrifugation using 40% Percoll. Single-cell suspensions were stained with anti-CD16/32 antibodies against surface molecules. For cell membrane protein staining, cells were stained at 4°C for 30 min. For intracellular cytokine staining, cells were incubated with stimulation cocktail for 4 h prior to cell surface and cytokine staining. Then, cell staining was performed after fixation and permeabilization with antibodies against the murine samples.
Instrument	BD Fortessa, BD FACSAria SORP and Cytek NL-3000
Software	BD FACSDiva Software and Cytek SpectroFlo Software were used to collect data, while Flowjo (10.8.1) was used to analyze flow cytometry data.
Cell population abundance	10,000 cells were analyzed for each sample. For sorting cell, the purity of cells was within post-sort fractions and regularly maintained > 95%.
Gating strategy	For all experiments, cells were first gated by FSC/SSC to exclude debris, followed by gating single cells. Then, target cell population for further analysis were gated by cell surface marker (e.g. CD45.2, CD3, NK1.1, CD8).

- [x] Tick this box to confirm that a figure exemplifying the gating strategy is provided in the Supplementary Information.
